# Editorial for E-Book: error awareness—insights from cognitive neuroscience, psychiatry and neurology

**DOI:** 10.3389/fnhum.2013.00830

**Published:** 2013-12-05

**Authors:** Tilmann A. Klein, Claudia Danielmeier, Markus Ullsperger

**Affiliations:** ^1^Department of Neurology, Max Planck Institute for Human Cognitive and Brain SciencesLeipzig, Germany; ^2^Day Clinic for Cognitive Neurology, University Clinic LeipzigLeipzig, Germany; ^3^Centre for Cognition, Donders Institute for Brain, Cognition, and Behaviour, Radboud University NijmegenNijmegen, Netherlands; ^4^Department of Neuropsychology, Institute of Psychology, Otto-von-Guericke University MagdeburgMagdeburg, Germany

**Keywords:** performance monitoring, error awareness, cognitive control, error-related negativity (ERN), error positivity (Pe), anterior cingulate cortex (ACC), insular cortex, consciousness

Most people would agree that error processing is essential for goal-directed and adaptive behavior. Within error processing one main distinction can be made between errors that become consciously aware and errors that remain unrecognized. Up until now it is still not completely understood what actually makes the difference between aware and unaware errors. Potential influences on error awareness can be manifold: endogenous factors like lack of attention or lack of expertise or exogenous factors like time pressure or ambiguous task situations. Some psychiatric or neurological diseases are at least at the surface also related to impaired processing of negative action outcomes.

Responses to action errors are not only restricted to brain responses: also vegetative changes can be observed (for example pupil diameter and heart rate). It is still matter of an ongoing debate whether these autonomous changes are cause or consequence of error awareness. Research on error awareness-related brain activity requires experimental paradigms with which error awareness can be manipulated. Given the many different influences on error awareness described above this is not an easy task.

Why should one investigate the distinction between aware and unaware errors then? Is it worthwhile that every error becomes aware? Or isn't it sometimes more economical that mistakes are going unrecognized? There are fields in human life in which high accuracy is necessary and errors need to be addressed: for example physicians should always be aware of irregularities within their professional routine. For this purpose it would of course be highly relevant to know about factors that promote error awareness. Whereupon it is an interesting question in itself whether it needs awareness/consciousness to trigger compensatory behavior.

Also from the clinical perspective it might be relevant to understand error awareness: either to improve awareness (for example in anosognosia) or to limit awareness (for example in obsessive-compulsive or affective disorders).

In order to tackle these practical/clinical questions basic issues need to be addressed: are there separate functional correlates of processing aware and unaware errors? Is there a relationship between electrophysiological correlates of conscious error processing (error-related negativity, ERN) and the electrophysiological response sensitive to error awareness (the error-positivity, PE)? Is there a continuum spanning from aware to unaware errors? And if so, is it just an accumulation of evidence that makes the difference between the two extremes? What cognitive tasks are actually suited best to investigate unaware errors?

The E-Book on error awareness tries to address some of these questions in form of review articles and original research papers. The authors cover different methodological (EEG/ fMRI, temporospatial/ICA analysis approaches), theoretical (ERN vs. PE, evidence accumulation, role of consciousness, saliency), clinical/pharmacological (schizophrenia, serotonin) and functional/structural anatomical (anterior cingulate cortex; insular cortex) topics. Taken together the articles cover large parts of current debate in error awareness research.

The E-Book starts with an article addressing the role of consciousness in cognitive control and decision making. van Gaal et al. (2012) review studies on unconscious information processing thereby showing that unconscious information is well capable of influencing different aspects of cognitive functioning or information processing. Sharpening the theoretical focus into the direction of error processing Harsay et al. (2012) show that there is indeed a functional overlap in brain structures for processes that are related on a theoretical level: (aware) error processing and processing of salient events in the environment. Especially the insular cortex, a region that will be mentioned in some other contributions, too, is engaged in both processes. Steinhauser and Yeung (2012) contribute another interesting theoretical consideration: they propose that error awareness is the result of evidence accumulation which in turn is reflected in an electrophysiological marker within the brain-electrical response to performance errors: the Pe. In his review, Wessel (2012) discusses the question whether error awareness is purely reflected in the error positivity or what the relation between the ERN and error awareness might be. Shalgi and Deouell (2012) follow a similar argument: they show that the amplitude of the ERN is indeed related to error awareness. They relate differences in error awareness to differences in actually reporting erroneous reactions and the confidence with which this report is made. The following two papers present different accounts of evaluating/assessing electrical brain responses to errors: Murphy et al. (2012) used independent component analysis to isolate the Pe within their EEG signal. This analysis enabled them to check for correlations between Pe amplitude and for example latency of the awareness-report response on single trial basis. Endrass et al. (2012) put forward a more spatially oriented analysis: they used principal component analysis in order to characterize spatial factors contributing to the Pe: a centro-parietal positivity correlated significantly with error awareness. Addressing error awareness with a higher spatial resolution Orr and Hester (2012) report data from functional magnetic resonance imaging (fMRI) showing that error-related activity in the dorsal anterior cingulate cortex is significantly higher in aware as compared to unaware errors. Most interestingly this area is often discussed as being one potential generator for the ERN thereby pointing back to the considerations of Wessel (2012) and Shalgi and Deouell (2012) reflecting on awareness influences on the ERN. The following three articles extend the focus of discussion to the fields of underlying neurochemical mechanisms, anatomical and clinical considerations. Mueller et al. (2012) showed that the neurotransmitter serotonin seems to be important for regulating the coupling between cortical and cardiac responses in performance monitoring. This seems especially relevant given the fact that the insular cortex is heavily involved in detecting bodily changes—a function which might contribute to error awareness. This central role of the Insula in error awareness is discussed in a review by Klein et al. (2013) with a special emphasis on anatomical considerations of Insula subdivisions and related functional aspects. Klein et al. (2013) also provide some information on pathological states that might alter error awareness and might as well be associated with changes in the insular cortex. Clinical aspects are rounded up by Núñez Castellar et al. (2012) showing that schizophrenic patients have more difficulties in reaching adequate performance levels in a performance monitoring task and that related adaptations in behavior following an error are reduced in these patients.

We believe that the collection of excellent scientific contributions gathered in this E-Book provides important new insights into the mechanisms and implications of conscious error perception. In addition, a number of outstanding questions can be derived from the reported findings, for example: Is error awareness a necessary and sufficient pre-requisite of post-error adjustments? Do post-error adjustments differ with respect to their association with error awareness? Can we infer on conscious error perception just based on brain data in the absence of interospective judgments? Which brain lesions and other neurological and psychiatric disorders interfere with error awareness? Hence, this E-Book can and should serve as the basis for new research lines in this field.
